# Additional prognostic value of polymorphisms within the 3′-untranslated region of programmed cell death pathway genes in early-stage breast cancer

**DOI:** 10.3389/fimmu.2024.1284579

**Published:** 2024-04-16

**Authors:** Hanxi Chen, Minyan Chen, Bangwei Zeng, Lili Tang, Qian Nie, Xuan Jin, Wenhui Guo, Lili Chen, Yuxiang Lin, Chuan Wang, Fangmeng Fu

**Affiliations:** ^1^ Department of Breast Surgery, Fujian Medical University Union Hospital, Fuzhou, Fujian, China; ^2^ Department of General Surgery, Fujian Medical University Union Hospital, Fuzhou, Fujian, China; ^3^ Breast Cancer Institute, Fujian Medical University, Fuzhou, Fujian, China; ^4^ Administration Department of Nosocomial Infection, Fujian Medical University Union Hospital, Fuzhou, Fujian, China; ^5^ Department of Pathology, Fujian Medical University Union Hospital, Fuzhou, Fujian, China

**Keywords:** breast cancer, survival, single nucleotide polymorphism, programmed cell death, prognosis

## Abstract

**Introduction:**

The programmed cell death (PCD) pathway plays an important role in restricting cancer cell survival and proliferation. However, limited studies have investigated the association between genetic variants in the 3′-untranslated region of the PCD pathway genes and breast cancer outcomes.

**Methods:**

In this study, we genotyped 28 potentially functional single nucleotide polymorphisms (SNPs) in 23 PCD pathway genes in 1,177 patients with early-stage breast cancer (EBC) from a Han Chinese population. The median follow-up period was 174 months.

**Results:**

Among all the candidate SNPs, four independent SNPs (rs4900321 and rs7150025 in ATG2B, rs6753785 in BCL2L11, and rs2213181 in c-Kit) were associated with invasive disease-free survival (iDFS), distant disease-free survival (DDFS), breast cancer-specific survival (BCSS) and overall survival (OS), respectively. Further combined genotypes of these four SNPs revealed that the survival decreased as the number of unfavorable genotypes increased (Ptrend = 1.0 × 10−6, 8.5 × 10−8, 3.6 × 10−4, and 1.3 × 10−4 for iDFS, DDFS, BCSS, and OS, respectively). Receiver operating characteristic curve analysis demonstrated that incorporating unfavorable genotypes and clinicopathological variables improved the ability to predict EBC survival (P = 0.006, 0.004, 0.029, and 0.019 for iDFS, DDFS, BCSS, and OS, respectively). Additionally, rs6753785 and rs2213181 were associated with BCL2L11 and c-Kit mRNA expression, respectively.

**Conclusions:**

Our results suggest that these four SNPs may act as novel biomarkers for EBC survival, possibly by modulating the expression of the corresponding genes.

## Introduction

Breast cancer is one of the major causes of cancer-related deaths among women in China and many other countries worldwide (1, 2). Notably, this disease is fundamentally and clinically heterogeneous, with variable survival outcomes. Although most patients with early-stage breast cancer are curable with current treatments, approximately 34% of patients who receive neoadjuvant or adjuvant chemotherapy die of breast cancer within 15 years (3). Biomarkers are required to predict which patients are at high risk of recurrence or metastasis. Moreover, inherited genetic variations may play a role in the prognosis of breast cancer, which has been verified by many candidate genes and genome-wide association studies (4–7).

MicroRNAs (miRNAs) are small, single-stranded, non-coding RNAs that modulate gene expression by binding to partially complementary sequences in the 3′-untranslated regions (3′-UTRs) of target mRNAs (8). Single nucleotide polymorphisms (SNPs), the most common genetic variations in the human genome, occur once every 100–300 base pairs. Recently, an increasing number of studies have demonstrated that SNPs within miRNA-binding regions contribute to cancer risk and outcomes by altering miRNA–mRNA binding affinities and miRNA-targeted gene expression (9–11).

Programmed cell death (PCD) is defined as regulated cell death executed by an intracellular program that includes several classic modalities, namely, apoptosis, autophagy, and programmed necrosis (12). Over the past decade, mounting evidence has suggested that these three types of programmed cell death are involved in cancer initiation and progression, making them promising pharmacological targets (12–14). miRNAs play important roles in regulating the PCD pathway and cancer progression (15, 16). However, few studies have investigated the association between the SNPs in the 3′-UTR of PCD-related genes and the susceptibility and prognosis of cancer (17–20).

Because of the requirements for sequence complementarity and stable thermodynamics around the miRNA–mRNA binding site, sequence variations within these regions are regarded as strong candidates for functional SNPs. Here, we hypothesized that genetic variations in miRNA–mRNA binding sites contribute to breast cancer recurrence and death. To test this hypothesis, we analyzed 28 SNPs in 3′-UTR of several key genes involved in PCD pathways with breast cancer prognosis in a large Han Chinese breast cancer cohort from Fuzhou, China.

## Materials and methods

### Study population

The patients were recruited from Fujian Medical University Union Hospital, Fujian, China, between July 2000 and October 2014. All 1,177 participants were Han Chinese women from Southeast China. All patients were diagnosed with early-stage (I–III) breast cancer and underwent curative surgical resection. Adjuvant treatments were chosen based on the surgical approach, patient’s menopausal status, and disease stage, in accordance with the relevant guidelines. Demographic and clinicopathological data were obtained from medical records. The estrogen receptor (ER), progesterone receptor (PR), and human epidermal growth factor-2 (HER2) statuses of each patient were evaluated using immunohistochemistry (IHC). All patients were followed up by personal or family contacts from the time of enrollment until death or the last follow-up (December 2016). The median follow-up period was 174 months. This study was approved by the Ethics Committee of the Fujian Medical University Union Hospital. All the participants provided written informed consent for inclusion in the study.

### SNP selection

Candidate genes of the PCD pathway were selected from the KEGG (http://www.genome.jp/kegg/) and miRDeathDB (http://rna-world.org/mirdeathdb/) databases. The 3′-UTR of candidate genes were selected from the UCSC genome browser (http://genome.ucsc.edu/). Furthermore, 28 SNPs in the 3′-UTR of PCD-related genes were extracted from miRBase (http://www.mirbase.org/), miRanda (http://www.microrna.org/), and TargetScans (http://www.targetscan.org/) with three filtering criteria: (1) minor allele frequency (MAF) must be ≥0.10 in the Han Chinese population in Beijing (CHB) from 1000 Genomes Project; (2) pairwise linkage disequilibrium between the eligible SNPs calculated by Haploview 4.1 software must be <0.8 (r^2^ < 0.8); and (3) SNPs were scored on the basis of the ΔΔG values, and only those that caused a change in the |ΔΔGtot| ≥ 2 kcal/mol (upper tertile) were considered biologically relevant and included in the study (21, 22). Information on eligible SNPs is presented in **Supplementary Table S1**.

### Genotyping

Blood samples were collected in tubes with ethylenediaminetetraacetic acid (EDTA) anticoagulant and stored at −80°C. Genomic DNA was extracted from EDTA anti-coagulated whole blood using a Whole-Blood DNA Extraction Kit (Bioteke, Beijing, China) according to the manufacturer’s protocol. DNA quality was assessed by agarose gel electrophoresis. Genotype analysis was performed using SNPScan, a high-throughput SNP genotyping tool (Genesky Biotechnologies Inc., Shanghai, China). Finally, raw data were analyzed using GeneMapper software (version 4.0; Applied Biosystems, Foster City, CA, USA). Five percent of the samples were randomly selected as blinded duplicates for quality assessment, and 100% concordance was obtained.

### Bioinformatics analysis

Expression quantitative trait locus (eQTL) analysis was performed for different tissue samples using the GTEx project portal (https://www.gtexportal.org/). Significant results were evaluated further. RegulomeDB (http://www.regulomedb.org) and HaploReg v4.2 (https://pubs.broadinstitute.org/mammals/haploreg/haploreg.php) were used to annotate potential functions of the selected SNPs. Differential gene expression analysis was performed using paired t-tests for paired samples. RNA-seq data of breast cancer tissues and corresponding adjacent normal tissues were downloaded from The Cancer Genome Atlas (TCGA) database (https://portal.gdc.cancer.gov). The Kaplan–Meier Plotter breast cancer microarray database (https://kmplot.com/analysis/index.php?p=service&cancer=breast) was used to assess the association between gene expression and breast cancer survival probability. Samples were divided into two groups according to median gene expression, and the JetSet best probe set was used for survival analysis.

### Statistical analysis

Overall survival (OS) and breast-cancer-specific survival (BCSS) were the primary endpoints of our study and were defined as the time from the date of cancer diagnosis to the date of all-cause mortality and death from breast cancer, respectively. Invasive disease-free survival (iDFS) and distant disease-free survival (DDFS) were the secondary endpoints and were calculated separately as the time from the date of diagnosis to the date of any invasive recurrence and distant relapse (23). Survival data were analyzed using the Kaplan–Meier (KM) method with the log-rank test and multivariate Cox stepwise regression analysis at the end of the follow-up (31 December 2016). Age at diagnosis, tumor size, lymph node involvement, histological grade, hormone receptor (HR) status, and HER-2/neu expression were adjusted. The hazard ratios (HRs) and 95% confidence intervals (CIs) for each factor in the multivariate analyses were calculated using a Cox regression model. Receiver operating characteristic (ROC) curve analysis was performed to estimate the area under the curve (AUC) of the logistic regression model. The Delong test was performed to compare the AUCs across different models. All tests were two-sided, and *p*-values <0.05 were considered statistically significant. SAS 9.4 (SAS Institute Inc., Cary, NC, USA) was used for all statistical analyses.

## Results

### Patients’ characteristics and clinical features

The clinical characteristics and survival information of the patients are summarized in **Table 1**. For the early-stage breast cancer (EBC) cohort, all cases were female patients, and their mean age was 47.0 ± 10.3 years at breast cancer diagnosis. During the follow-up period, 446 patients experienced recurrence (446 locoregional and 410 distant) and 343 died (333 died of BC and 10 from other diseases).

**Table 1:** Clinicopathological characteristics and clinical outcome of EBC patients.

Variables	Patients^a^ N=1,177	iDFS		DDFS		BCSS		OS	
		Events	LogRank *p*	Events	LogRank *p*	Events	LogRank *p*	Events	LogRank *p*
Age at diagnosis			0.021		0.087		0.420		0.402
≤ 35	184	85		76		59		61	
> 35	993	361		334		274		282	
Tumor size			<0.001		<0.001		<0.001		<0.001
≤ 2cm	403	88		80		67		70	
>2cm	774	358		330		266		273	
Nodal status			<0.001		<0.001		<0.001		<0.001
negative	510	116		101		69		75	
positive	667	330		309		264		268	
Clinical stage			<0.001		<0.001		<0.001		<0.001
I	257	40		35		29		31	
II+III	920	406		375		304		312	
Grade			<0.001		<0.001		<0.001		<0.001
I+II	904	310		286		228		236	
III	271	134		122		103		105	
ER			<0.001		<0.001		<0.001		<0.001
Negative	378	177		165		149		150	
Positive	799	269		245		184		193	
HR			<0.001		<0.001		<0.001		<0.001
Negative	367	171		159		144		145	
Positive	810	275		251		189		198	
HER2			<0.001		<0.001		<0.001		<0.001
Negative	860	292		268		214		222	
Positive	317	154		142		119		121	
Subtype			<0.001		<0.001		<0.001		<0.001
Luminal A	236	35		33		26		26	
Luminal B	574	240		218		163		172	
HER2 +	160	80		76		67		67	
Triple negative	207	91		83		77		78	
Chemotherapy			0.901		0.900		0.209		0.350
No	69	28		26		17		19	
Yes	1,108	418		384		316		324	
Endocrine therapy			<0.001		<0.001		<0.001		<0.001
No	387	189		175		153		156	
Yes	789	257		235		180		187	
HER2-targeted therapy			0.036		0.185		0.538		0.626
No	1,125	422		390		318		328	
Yes	52	24		20		15		15	

a Variable including missing data.

Patients with tumor size >2 cm, lymph node positivity, clinical stage II + IIIs, or grade III had worse survival (log-rank *p* < 0.001). HER2 positivity was also associated with a short survival time, whereas ER/HR-positive and luminal A subtypes were associated with improved survival (log-rank *p <*0.001). However, no association was observed between the age at diagnosis and DDFS, BCSS, or OS (log-rank *p* = 0.087, 0.420, and 0.402, respectively).

### Effects of SNPs in 3′-UTR of PCD-related pathway genes on EBC survival

The effects of different genotype distributions for each SNP on the survival of patients with early-stage breast cancer were evaluated using the log-rank test. Three SNPs (rs4900321, rs7150025, and rs6753785) in the dominant model were significantly associated with iDFS, DDFS, BCSS, and OS in patients with EBC (all log-rank *p* < 0.05, **Table 2**).

**Table 2:** Genotyping results with early breast cancer survival.

SNPs	Cases WH/H/VH	iDFS (LogRank *p*)				DDFS (LogRank *p*)				BCSS (LogRank *p*)				OS (LogRank *p*)			
		Events WH/H/VH	DOM	REC	ADD	Events WH/H/VH	DOM	REC	ADD	Events WH/H/VH	DOM	REC	ADD	Events WH/H/VH	DOM	REC	ADD
rs1042542	489/541/153	182/193/70	0.912	0.037	0.082	169/175/65	0.775	0.047	0.088	138/143/51	0.646	0.161	0.239	142/147/53	0.646	0.136	0.204
rs1052550	903/238/33	343/91/10	0.920	0.386	0.675	312/87/9	0.768	0.384	0.570	247/76/8	0.298	0.688	0.435	255/78/8	0.324	0.622	0.432
rs1059234	294/578/303	125/203/117	0.818	0.558	0.224	119/185/105	0.061	0.890	0.123	90/155/87	0.489	0.764	0.669	95/158/89	0.322	0.810	0.507
rs12082	930/236/11	355/89/2	0.518	0.127	0.296	323/85/2	0.903	0.171	0.386	258/73/2	0.648	0.281	0.436	264/77/2	0.442	0.255	0.304
rs1530865	722/403/51	266/163/16	0.564	0.264	0.349	247/146/16	0.757	0.471	0.670	206/113/13	0.643	0.534	0.787	211/118/13	0.746	0.459	0.755
rs205107	661/455/60	257/167/21	0.398	0.708	0.691	242/150/17	0.128	0.334	0.265	193/121/18	0.337	0.679	0.500	198/126/18	0.373	0.792	0.591
rs205108	437/573/166	172/213/60	0.456	0.828	0.757	162/192/55	0.219	0.816	0.464	133/148/51	0.230	0.349	0.192	135/155/52	0.341	0.393	0.309
rs2213181	997/168/12	381/58/7	0.524	0.076	0.122	354/49/7	0.248	0.058	0.046	285/42/6	0.580	0.089	0.148	295/42/6	0.423	0.105	0.132
rs2239680	691/417/69	263/153/30	0.980	0.327	0.595	240/140/30	0.887	0.123	0.290	197/111/25	0.968	0.128	0.276	203/115/25	0.988	0.172	0.357
rs2285332	568/475/107	200/193/44	0.040	0.449	0.121	188/174/39	0.188	0.733	0.420	152/148/27	0.204	0.446	0.207	153/155/29	0.080	0.599	0.111
rs2459965	912/255/10	350/94/2	0.482	0.247	0.452	320/88/2	0.637	0.307	0.569	260/72/1	0.578	0.218	0.442	268/74/1	0.542	0.205	0.415
rs3088440	905/258/13	352/91/3	0.250	0.319	0.380	327/80/3	0.140	0.428	0.294	265/65/3	0.228	0.698	0.477	274/66/3	0.169	0.659	0.383
rs3213180	503/553/121	188/210/48	0.830	0.644	0.896	175/188/47	0.998	0.274	0.522	139/157/37	0.639	0.443	0.720	143/161/39	0.596	0.325	0.596
rs35592567	804/337/36	304/127/15	0.990	0.549	0.824	280/118/12	0.864	0.947	0.985	220/102/11	0.435	0.651	0.712	226/106/11	0.388	0.738	0.684
rs3751711	780/357/39	304/129/13	0.416	0.510	0.647	278/120/12	0.532	0.483	0.706	222/102/9	0.971	0.389	0.676	229/105/9	0.931	0.334	0.616
rs4252745	622/481/74	243/178/25	0.318	0.434	0.529	225/163/22	0.288	0.362	0.462	185/129/19	0.246	0.631	0.503	192/132/19	0.176	0.532	0.386
rs4746720	385/575/215	141/221/83	0.370	0.762	0.670	132/200/77	0.716	0.783	0.924	104/164/64	0.534	0.632	0.791	107/170/65	0.529	0.734	0.812
rs4789560	395/608/173	162/220/63	0.101	0.784	0.256	149/205/55	0.154	0.471	0.344	120/165/47	0.283	0.812	0.560	124/169/49	0.249	0.877	0.506
rs4900321	795/346/36	271/159/16	0.000	0.320	0.001	243/152/15	0.000	0.329	0.000	207/116/10	0.021	0.955	0.059	211/122/10	0.008	0.964	0.022
rs6753785	405/569/203	175/198/73	0.004	0.519	0.015	165/179/66	0.002	0.419	0.008	132/146/55	0.016	0.624	0.052	136/150/57	0.014	0.664	0.046
rs6861	295/534/346	111/205/130	0.794	0.800	0.952	103/189/118	0.964	0.915	0.994	81/152/100	0.665	0.417	0.712	82/158/103	0.531	0.416	0.675
rs701848	415/541/221	156/198/92	0.972	0.263	0.497	146/180/84	0.781	0.372	0.549	117/150/66	0.861	0.734	0.898	123/152/68	0.620	0.714	0.749
rs7150025	693/405/77	287/134/24	0.002	0.265	0.007	268/119/22	0.000	0.324	0.003	215/101/17	0.010	0.287	0.034	222/104/17	0.007	0.224	0.023
rs72822657	715/418/42	279/153/14	0.354	0.449	0.561	252/146/12	0.666	0.339	0.620	202/124/7	0.937	0.087	0.217	210/126/7	0.736	0.071	0.194
rs73500020	831/315/28	312/116/18	0.748	0.004	0.015	289/104/17	0.966	0.006	0.018	240/85/8	0.411	0.914	0.710	246/89/8	0.468	0.834	0.768
rs8116	362/563/251	137/207/101	0.983	0.451	0.729	124/191/95	0.693	0.370	0.667	106/155/72	0.784	0.975	0.954	107/159/77	0.983	0.674	0.907
rs884225	327/581/268	120/225/101	0.777	0.633	0.798	115/210/85	0.764	0.135	0.319	88/175/70	0.622	0.236	0.312	89/182/72	0.470	0.220	0.223
rs9552315	310/597/270	112/230/104	0.582	0.987	0.846	109/207/94	0.737	0.844	0.941	92/169/72	0.378	0.409	0.576	92/177/74	0.608	0.375	0.656

WH, wild-type homozygote; H, heterozygote; VH, variant homozygote; DOM, dominant model; REC, recessive model; ADD, additive model.

Multivariate Cox regression analyses revealed that four SNPs (rs4900321 and rs7150025 in *ATG2B*, rs6753785 in *BCL2L11*, and rs2213181 in *c-Kit*) were significantly associated with EBC prognosis in the different genetic models (**Table 3**). Among these four SNPs, rs4900321and rs2213181 were associated with worse EBC survival (rs4900321 in the dominant model: aHR = 1.330, *p* = 0.004 for iDFS; 1.444, 0.0003 for DDFS; and 1.268, 0.034 for OS; rs2213181 in the recessive model: aHR = 3.034, *p* = 0.004 for iDFS; 3.118, 0.003 for DDFS; 3.088, 0.007 for BCSS; and 2.919, 0.010 for OS), while rs6753785 and rs7150025 were associated with better survival (rs7150025 in dominant model: aHR = 0.719, *p* = 0.0009 for iDFS; 0.706, 0.008 for DDFS; 0.751, 0.013 for BCSS; and 0.741, 0.009 for OS; rs6753785 in dominant model: aHR = 0.748, *p* = 0.006 for iDFS; 0.729, 0.002 for DDFS; 0.755, 0.013 for BCSS; and 0.757, 0.013 for OS).

**Table 3:** Association between the SNPs genotypes and early breast cancer survival (multivariate cox proportional hazard model).

SNPs	Cases	iDFS			DDFS			BCSS			OS		
		Events	Adjusted HR^a^	*p*-value	Events	Adjusted HR^a^	*p*-value	Events	Adjusted HR^a^	*p*-value	Events	Adjusted HR^a^	*p*-value
rs4900321													
AA	795	271	1 (reference)		243	1 (reference)		207	1 (reference)		211	1 (reference)	
AT	346	159	1.317 (1.080–1.605)	0.006	152	1.433 (1.168–1.759)	0.0006	116	1.239 (0.985–1.560)	0.067	122	1.287 (1.027–1.613)	0.028
TT	36	16	1.482(0.890–2.467)	0.130	15	1.567 (0.925–2.655)	0.095	10	1.083 (0.572–2.052)	0.807	10	1.072 (0.566–2.029)	0.832
DOM			1.330 (1.098–1.612)	0.004		1.444 (1.083–1.763)	0.0003		1.225 (0.979–1.532)	0.075		1.268 (1.017–1.579)	0.034
REC			1.350 (0.816–2.236)	0.243		1.384 (0.822–2.331)	0.221		1.010 (0.536–1.903)	0.976		0.986 (0.523–1.857)	0.965
rs6753785													
GG	405	175	1 (reference)		165	1 (reference)		132	1 (reference)		136	1 (reference)	
GT	569	198	0.757 (0.625–0.918)	0.005	179	0.725 (0.586–0.890)	0.003	146	0.744 (0.586–0.944)	0.015	150	0.743 (0.587–0.940)	0.013
TT	203	73	0.923 (0.716–1.188)	0.533	66	0.887 (0.680–1.157)	0.041	55	0.786 (0.572–1.080)	0.137	57	0.795 (0.582–1.087)	0.668
DOM			0.748 (0.610–0.919)	0.006		0.729 (0.597–0.890)	0.002		0.755 (0.604–0.943)	0.013		0.757 (0.608–0.942)	0.013
REC			0.783 (0.594–1.031)	0.082		0.887 (0.680–1.157)	0.377		0.927 (0.692–1.243)	0.614		0.939 (0.705–1.252)	0.151
rs7150025													
GG	693	287	1 (reference)		268	1 (reference)		215	1 (reference)		222	1 (reference)	
GT	405	134	0.727 (0.591–0.894)	0.002	119	0.708 (0.570–0.880)	0.002	101	0.766 (0.604–0.973)	0.029	104	0.760 (0.601–0.962)	0.022
TT	77	24	0.675 (0.441–1.033)	0.071	22	0.692 (0.444–1.080)	0.266	17	0.640 (0.385–1.064)	0.117	17	0.640 (0.385–1.064)	0.085
DOM			0.719 (0.591–0.874)	0.0009		0.706 (0.575–0.866)	0.008		0.751 (0.598–0.942)	0.013		0.741 (0.593–0.927)	0.009
REC			0.755 (0.496–1.150)	0.191		0.779 (0.502–1.210)	0.266		0.729 (0.441–1.206)	0.219		0.704 (0.426–1.163)	0.171
rs2213181													
CC	997	381	1 (reference)		354	1 (reference)		285	1 (reference)		295	1 (reference)	
CT	168	58	0.823 (0.624–1.085)	0.167	49	0.748 (0.555–1.009)	0.057	42	0.821 (0.593–1.136)	0.233	42	0.789 (0.571–1.091)	0.151
TT	12	7	2.948 (1.386–6.274)	0.005	7	2.992 (1.408–6.361)	0.004	6	2.999 (1.326–6.783)	0.008	6	2.820 (1.248–6.375)	0.013
DOM			0.892 (0.685–1.161)	0.394		0.825 (0.622–1.094)	0.182		0.902 (0.664–1.226)	0.509		0.866 (0.638–1.176)	0.358
REC			3.034 (1.427–6.449)	0.004		3.118 (1.468–6.621)	0.003		3.088 (1.367–6.977)	0.007		2.919 (1.293–6.592)	0.010
Combined^b^													
0–1	705	223	1 (reference)		196	1 (reference)		168	1 (reference)		171	1 (reference)	
2	363	168	1.614 (1.318–1.975)	3.5E−6	162	1.803 (1.461–2.225)	3.8E−8	124	1.508 (1.194–1.905)	5.6E−4	130	1.568 (1.246–1.973)	1.2E−4
3–4	109	55	1.703 (1.266–2.292)	4.3E−4	52	1.777 (1.306–2.417)	2.5E−4	41	1.562(1.108–2.203)	0.011	42	1.579(1.124–2.218)	0.008
Trend *p*			1.381 (1.213–1.572)	1.0E−6		1.437 (1.259–1.641)	8.5E−8		1.315(1.131–1.528)	3.6E−4		1.334 (1.151–1.546)	1.3E−4

DOM, dominant model; REC, recessive model.

a Adjusted for age at diagnosis, tumor size, lymph node involvement, grade, hormone receptor, and HER2 status.

b The unfavorable genotypes were defined as rs4900321-AT+TT, rs6753785 GG, rs7150025 GG, and rs2213181 TT.

### Stratified analysis for association between SNPs and EBC survival

To further explore whether the association between SNP genotypes and EBC survival was modified by other variables, an analysis stratified according to age at diagnosis, tumor size, lymph node involvement, grade, hormone receptor status, and HER2 status was performed. As shown in **Supplementary Table S2**, we observed the association between rs4789560 and worse survival among young patients (rs4789560 for age at diagnosis ≤ 35: aHR = 1.464, 95% CI = 0.863–2.484 for DDFS, 1.722, 0.909–3.262 for BCSS, and 1.871, 0.988–3.544 for OS). Furthermore, analysis stratified by HER2 status revealed that rs2285332 and rs205107 were associated with survival in patients with higher-grade tumors (rs2285332 for grade III: aHR = 2.364, 95% CI = 1.275–4.383 for iDFS; 2.383, 1.249–4.548 for DDFS; 2.477, 1.215–5.050 for BCSS; and 2.391, 1.174–4.869 for OS; rs205107 for grade III: aHR = 2.258, 95% CI = 1.043–4.889 for iDFS, 1.844, 0.848–4.011 for DDFS; 2.621, 1.186–5.790 for BCSS, and 2.479, 1.126–5.459 for OS). Moreover, rs4900321 in *ATG2B* was found to have a detrimental effect, while rs7150025 in *ATG2B* had a beneficial effect on survival in HER2-positive patients (rs4900321 for HER2 positive: aHR = 1.747, 95% CI = 1.266–2.413 for iDFS; 2.013, 1.442–2.809 for DDFS; 1.827, 1.269–2.630 for BCSS; and 1.860, 1.296–2.669 for OS; rs7150025 for HER2 positive: aHR = 0.472, 95% CI = 0.330–0.676 for iDFS; 0.496, 0.341–0.721 for DDFS; 0.493, 0.327–0.744 for BCSS; and 0.500, 0.333–0.751 for OS). However, no obvious differences in the effects of the SNPs on EBC survival were observed among the other strata.

### Prognostic significance of risk genotypes in different molecular subtypes of EBC

Next, we performed a subgroup analysis according to the IHC-based breast cancer subtypes. Several variants were found to be strongly associated with HER2 enriched and Luminal B EBC outcomes (**Table 4**). HER2-enriched patients with the AT + TT genotype for rs4900321 had worse survival (aHR = 1.870, *p* = 0.006 for iDFS; 2.115, 0.001 for DDFS; 2.076, 0.003 for BCSS; and 2.076, 0.003 for OS) than did patients carrying the AA genotype. In contrast, rs7150025 in the dominant model was significantly correlated with better prognosis (aHR = 0.394, *p* = 0.0005 for iDFS; 0.422, 0.002 for DDFS; 0.388, 0.001 for BCSS; and 0.388, 0.001 for OS). Moreover, we found that rs2213181 was markedly associated with survival in Luminal B early-stage breast cancer. The TT carriers for rs2213181 had worse survival than did the CC/CT carriers (aHR = 3.920, *p* = 0.008 for iDFS; 5.017, 0.002 for DDFS; 8.541, 3.7×10^−5^ for BCSS; and 7.428, 0.0001 for OS).

**Table 4:** Association between the SNPs genotypes and breast cancer survival based on subtypes (multivariate cox proportional hazard model).

SNPs	Cases	iDFS			DDFS			BCSS			OS		
		Events	Adjusted HR^a^	*p-*value	Events	Adjusted HR^a^	*p-*value	Events	Adjusted HR^a^	*p-*value	Events	Adjusted HR^a^	*p-*value
HER2 enrich													
rs4900321													
AA	107	45	1 (reference)		41	1 (reference)		36	1 (reference)		36	1 (reference)	
AT	48	30	1.689 (1.061–2.689)	0.027	30	1.904 (1.186–3.058)	0.008	27	1.966 (1.191–3.244)	0.008	27	1.966 (1.191–3.244)	0.008
TT	5	5	6.399 (2.435–16.81)	0.0001	5	8.003 (3.005–21.31)	3.2E−5	4	3.493 (1.190–10.26)	0.023	4	3.493 (1.190–10.26)	0.023
DOM			1.870 (1.198–2.921)	0.006		2.115 (1.342–3.332)	0.001		2.076 (1.280–3.366)	0.003		2.076 (1.280–3.366)	0.003
REC			5.236 (2.035–13.47)	0.006		6.222 (2.395–16.16)	0.0002		2.732 (0.952–7.838)	0.062		2.732 (0.952–7.838)	0.062
rs7150025													
GG	102	61	1 (reference)		58	1 (reference)		52	1 (reference)		52	1 (reference)	
GT	46	16	0.402 (0.230–0.703)	0.001	15	0.423 (0.239–0.752)	0.003	13	0.404 (0.219–0.745)	0.004	13	0.404 (0.219–0.745)	0.004
TT	12	3	0.357 (0.112–1.143)	0.083	3	0.413 (0.129–1.325)	0.137	2	0.309 (0.075–1.273)	0.104	2	0.309 (0.075–1.273)	0.104
DOM			0.394 (0.234–0.664)	0.0005		0.422 (0.247–0.719)	0.002		0.388(0.217–0.691)	0.001		0.388(0.217–0.691)	0.001
REC			0.465 (0.146–1.478)	0.194		0.526 (0.165–1.674)	0.277		0.398 (0.097–1.631)	0.200		0.398 (0.097–1.631)	0.200
Luminal B													
rs2213181													
CC	493	203	1 (reference)		186	1 (reference)		136	1 (reference)		145	1 (reference)	
CT	76	33	0.949 (0.655–1.374)	0.781	28	0.851 (0.571–1.269)	0.429	23	0.974 (0.634–1.521)	0.907	23	0.917 (0.588–1.428)	0.700
TT	5	4	3.896 (1.430–10.62)	0.008	4	4.926 (1.801–13.48)	0.002	3	8.514 (3.069–23.63)	3.91E−5	3	7.352 (2.661–20.32)	0.0001
DOM			1.036 (0.728–1.475)	0.842		0.953 (0.653–1.390)	0.802		1.128 (0.743–1.711)	0.572		1.059 (0.700–1.603)	0.786
REC			3.920 (1.440–10.67)	0.008		5.017 (1.835–13.71)	0.002		8.541 (3.082–23.67)	3.70E−5		7.428 (2.691–20.50)	0.0001

DOM, dominant model; REC, recessive model.

a Adjusted for age at diagnosis, tumor size, lymph node involvement, grade, hormone receptor, and HER2 status.

### Combined analysis of four independent SNPs on survival of EBC

**Figure 1:**
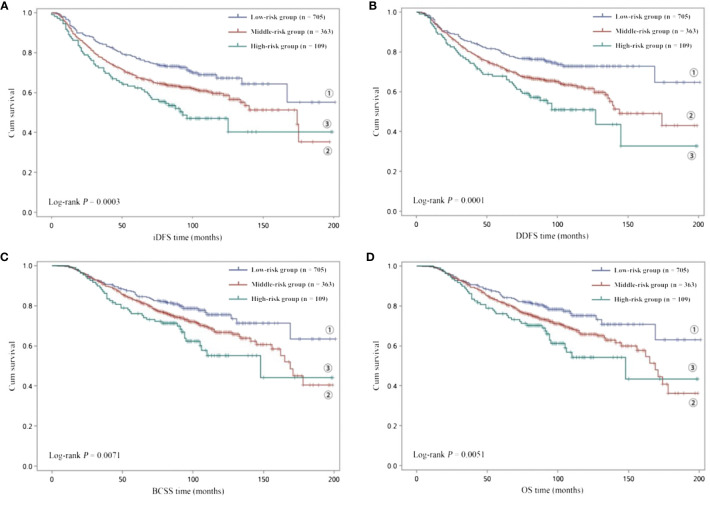
Kaplan–Meier analysis of **(A)** iDFS and **(B)** DDFS and **(C)** BCSS and **(D)** OS for low-risk (0 unfavorable genotype), middle-risk (1–2 unfavorable genotypes) and high-risk (3–4 unfavorable genotypes) EBC patients.

Subsequently, to investigate the potential combined effects of the four independent SNPs on EBC survival, we combined the risk genotypes of rs4900321 AT+TT, rs6753785 GG, rs7150025 GG, and rs2213181 TT into a single variable as the number of unfavorable genotypes. We divided the combined unfavorable genotypes into three groups: low risk (0 unfavorable genotypes), middle risk (1–2 unfavorable genotypes), and high risk (3–4 unfavorable genotypes). Kaplan–Meier analysis revealed that survival, including iDFS, DDFS, BCSS, and OS, decreased as the number of risk genotypes increased (log-rank *p* = 0.0003 for iDFS; 0.0001 for DDFS; 0.0071 for BCSS; and 0.0051 for OS; **Figure 1**). Compared with the low-risk group in the multivariate analysis, the middle- and high-risk groups were associated with decreasing risks of recurrence and death in a dose-dependent manner (aHR = 1.381, adjusted *p*_trend_ = 1.0×10^−6^ for iDFS; 1.437, 8.5×10^−8^ for DDFS; 1.315, 3.6×10^−4^ for BCSS; and 1.334, 1.3×10^−4^ for OS; **Table 3**).

### Survival predictive model based on clinical factors combined with risk genotypes

**Figure 2:**
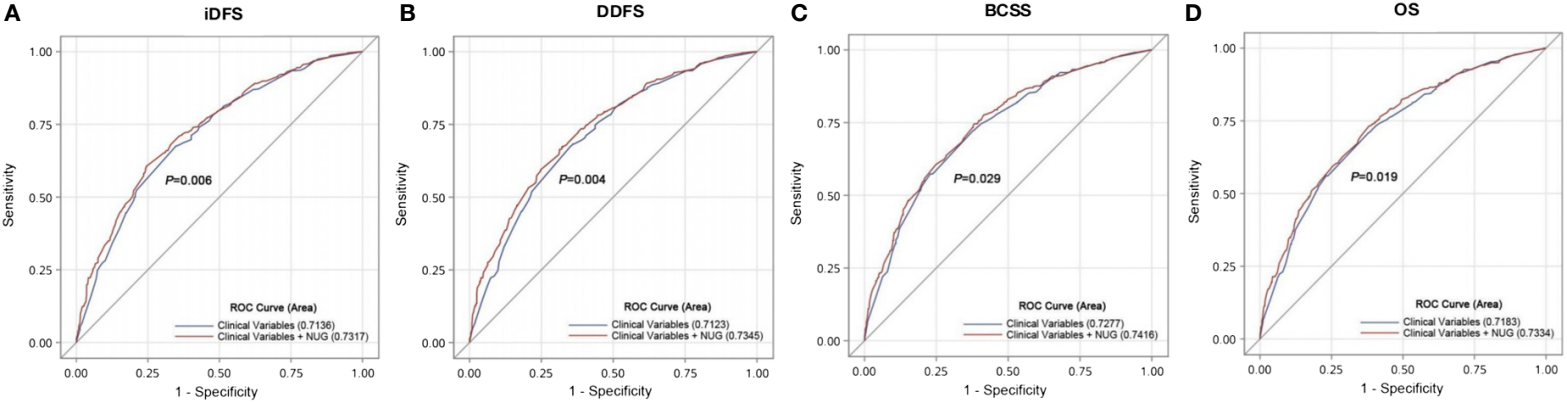
Validation of the prognostic model that combines risk genotypes and clinical characteristics to predict EBC survival. ROC curves and AUCs were used to assess the prognostic accuracy for **(A)** iDFS and **(B)** DDFS and **(C)** BCSS and **(D)** OS.

We constructed a prognostic model that combined risk genotypes and clinical characteristics for survival. The predictive value of the model was assessed using an ROC curve analysis. Compared with the reference model of clinical factors, including age at diagnosis, tumor size, lymph node involvement, grade, hormone receptor, and HER2 status, the model with the addition of unfavorable genotypes improved the predictive capability for iDFS (AUC = 0.732, 95% CI = 0.702–0.761, *p* = 0.006, **Figure 2A**), DDFS (AUC = 0.735, 95% CI = 0.705–0.764, *p* = 0.004, **Figure 2B**), BCSS (AUC = 0.742, 95% CI = 0.710–0.773, *p* = 0.029, **Figure 2C**), and OS (AUC = 0.733, 95% CI = 0.702–0.765, *p* = 0.019, **Figure 2D**).

### eQTL analysis and functional prediction

Next, we sought to better understand the potential mechanisms by which these SNPs influenced the prognosis of EBC. The GTEx Portal eQTL Browser was used to evaluate the correlations between these four SNPs and the mRNA expression of their corresponding genes (**Supplementary Table S3**). We found that the rs2213181C allele correlated with heightened expression levels of *c-Kit* in normal breast tissues (*p* = 0.023). The rs6753785T allele was significantly associated with increased *BCL2L11* expression levels in brain tissues (*p* < 0.001) but not in normal breast tissues (*p* = 0.9). rs4900321 and rs7150025 statuses had no significant correlation with *ATG2B* mRNA expression levels (*p* = 0.81 and 0.47, respectively).

We then performed functional predictions for these four identified SNPs using the online bioinformatics databases RegulomeDB and Haploreg. The results showed that these four SNPs affected promoter histone marks, enhancer histone marks, DNase, and motifs. The details of the biological function predictions are summarized in **Supplementary Table S4**.

### Differential mRNA expression analysis

Finally, we analyzed the mRNA expression of *ATG2B*, *BCL2L11*, and *c-Kit* in 113 pairs of breast tumor and adjacent normal tissue samples obtained from the TCGA database. Kaplan–Meier survival analysis was used to assess the association between the expression levels of these genes and the probability of breast cancer survival. Survival data were obtained from the Kaplan–Meier plotter database. Compared to adjacent normal tissues, breast cancer tissues had lower mRNA expression levels of *ATG2B* and *c-Kit* (*p <*0.001 for both; **Supplementary Figures S1A, C**). Moreover, lower expression levels of *ATG2B* and *c-Kit* mRNA were significantly associated with worse breast cancer recurrence-free survival (RFS) and OS (all log-rank *p <*0.001; **Supplementary Figures S1D, F, G, I**). Although the expression levels of *BCL2L11* were higher in breast cancer tissues compared to normal breast tissues (*p* < 0.001, **Supplementary Figure S1B**), patients with higher *BCL2L11* expression within the breast cancer cohort exhibited better RFS and OS compared to those with lower *BCL2L11* expression (log-rank *p* = 0.0016 and 0.013, respectively; **Supplementary Figures S1E, H**).

## Discussion

In this study, we conducted a clinical follow-up to systematically investigate the correlation between miRNA-binding site polymorphisms in PCD pathway genes and disease progression and death in patients with EBC. We found that the variant genotypes *ATG2B* rs4900321AT+TT and rs7150025 GG, *BCL2L11* rs6753785 GG, and *c-Kit* rs2213181 TT or a combination of these genotypes was significantly associated with an increased risk of EBC survival. Additionally, we discovered that several polymorphisms might play important roles in the progression of HER2 enriched (*ATG2B* rs4900321 and rs7150025) and Luminal B (*c-Kit* rs2213181) early breast cancer. Further bioinformatics analyses suggested that the rs6753785T and rs2213181C alleles were correlated with higher *BCL2L11* and *c-Kit* expression.

*ATG2B* is a core member of the autophagy-related gene (ARG). Autophagy has been reported to play a dual role in tumor development. *ATG2B* is a direct target of miR-130a, which inhibits autophagy and promotes cell death and chemosensitivity by downregulating *ATG2B* expression in chronic lymphocytic leukemia and gastrointestinal stromal tumor (GIST) (24, 25). Conversely, ATG2B has been identified as a potential protective biomarker in Ewing’s sarcoma (26). In a recent study by Park et al., ATG2B was shown to inhibit cancer stemness in TNBC (27). Moreover, Zhang et al. found that decreased transcription and expression of *ATG2B* was related to ER/PR-negative breast cancer, and methylation of *ATG2B* was also correlated with high-grade and TNM stage in invasive ductal carcinomas (28). According to the KM plotter database, higher *ATG2B* mRNA expression is associated with better RFS and OS in patients with breast cancer. Our genetic association study indicated that putative miRNAs binding site rs4900321 and rs7150025, located at the 3′-UTR of *ATG2B*, had an independent impact on the risk of EBC recurrence and death, particularly in HER2-positive breast cancer. We speculate that the rs4900321T and rs7150025G alleles could modify EBC outcomes by influencing the binding affinity of miRNAs and altering *ATG2B* expression. Further biological and functional studies are needed to determine the role of rs4900321 and rs7150025 in *ATG2B* during EBC progression.

c-Kit, also known as CD117, is a type III transmembrane receptor protein kinase that regulates cellular proliferation, differentiation, adhesion, and apoptosis. Notably, c-Kit-positive ovarian cancer cells showed higher autophagy levels (29), and c-Kit has been identified as an oncogenic driver and prognostic risk factor for various malignancies, including GIST, osteosarcoma, and ovarian cancer (30–32); however, its role in breast cancer remains unclear. C-Kit overexpression, assessed by immunohistochemistry, has been proven to be an independent indicator of worse prognosis in breast carcinomas and basal-like breast cancer (33, 34). However, high c-Kit expression levels occur infrequently in breast cancer, and *c-Kit* somatic gene mutations are absent in breast cancer (35). Tsutsui et al. reported that low c-Kit expression is significantly correlated with lymph node metastasis and worse survival in invasive breast cancer (36). Another study demonstrated that TNBC patients with at least one positive expression of three biomarkers (c-Kit, CK5, and EGFR) had a longer OS (37). With respect to common variants in *c-Kit* gene, a few studies have revealed that some SNPs in *c-Kit* may be predisposed to melanoma and GIST (38, 39). In our study, we found that the rs2213181T allele was associated with low *c-Kit* mRNA expression levels and worse prognosis in EBC, especially Luminal B EBC. Additionally, survival data from the KM plotter database indicated a favorable effect of *c-Kit* expression on breast cancer survival. Similarly, rs2213181 was associated with non-small-cell lung cancer (NSCLC) survival and acute myeloid leukemia susceptibility (40, 41). We speculated that rs2213181 might regulate the expression of *c-Kit* genes and influence EBC progression. However, the molecular mechanisms by which c-KIT regulates breast cancer cell proliferation remain unclear.

BCL2L11 (also known as BIM) is an essential proapoptotic BH3-only protein that initiates the intrinsic apoptotic pathway and plays an important role in promoting apoptosis in many cancer cells (42). Specifically, BCL2L11 is a tumor suppressor that regulates cell proliferation, metastasis, and chemotherapeutic resistance in breast cancer (43, 44). Genetic variations in *BCL2L11* mediate the heterogeneity of responses to tyrosine kinase inhibitors in NSCLC and chronic myeloid leukemia (45, 46). In addition, rs71801447, which lies in the 3′-UTR of *BCL2L11*, was associated with *BCL2L11* expression and anti-tumor immune response in breast cancer (47). The above experimental evidence suggests that variants in *BCL2L11* may be of great importance to the susceptibility and treatment response of some cancers. In our study, the rs6753785G allele was a poor prognostic indicator of EBC and was significantly associated with lower *BCL2L11* mRNA expression in some specific tissues. These results support the hypothesis that rs6753785 influences EBC prognosis by altering *BCL2L11* expression.

However, some potential limitations should be taken into consideration. First, there were geographical limitations because all participants were recruited from the same hospital. Second, it is difficult to explore gene–environment interactions in the development of breast cancer because of the absence of relevant information. Finally, although several genetic variants in the 3′-UTR of PCD-related genes were found to be associated with EBC survival based on *in silico* analyses, the biological mechanisms underlying the observed association between these SNPs and the development and progression of EBC remain unclear.

In conclusion, this study revealed that rs4900321 and rs7150025 in *ATG2B*, rs6753785 in *BCL2L11*, and rs2213181 in *c-Kit* are associated with early breast cancer prognosis in a Chinese population. These potential functional loci could be used as biomarkers to identify patients with EBC at a high risk of recurrence or death who require more aggressive treatment. Further studies will focus on the evaluation of the 3′-UTR SNP-induced miRNA targeted gene regulation and the functional role of these genes in breast cancer.

## Data availability statement

The original contributions presented in the study are included in the article/**Supplementary Material**, further inquiries can be directed to the corresponding author/s.

## Ethics statement

The studies involving humans were approved by Research Ethics Committee of Fujian Medical University Union Hospital. The studies were conducted in accordance with the local legislation and institutional requirements. Written informed consent was obtained from the patients/participants.

## Author contributions

HC: Conceptualization, Investigation, Resources, Writing – original draft. MC: Funding acquisition, Investigation, Resources, Validation, Writing – original draft. BZ: Methodology, Writing – review & editing. LT: Resources, Validation, Writing – review & editing. QN: Resources, Writing – review & editing. XJ: Investigation, Writing – review & editing. WG: Writing – review & editing. LC: Funding acquisition, Writing – review & editing. YL: Conceptualization, Resources, Supervision, Writing – review & editing. CW: Funding acquisition, Project administration, Supervision, Writing – review & editing. FF: Conceptualization, Funding acquisition, Project administration, Supervision, Writing – review & editing.
