# Supplementary material for: Additional prognostic value of polymorphisms within the 3′-untranslated region of programmed cell death pathway genes in early-stage breast cancer
Source: Front Immunol. 2024 Apr 16;15:1284579. doi: 10.3389/fimmu.2024.1284579 (PMC11058218; doi:10.3389/fimmu.2024.1284579)
Supplement: Supplementary file 1 [file DataSheet_1.pdf]

Fig.S1 Differential mRNA expression analysis (a-c) and Kaplan-Meier survival analysis (d-i) of the three genes. (a) *ATG2B*, (b) *BCL2L11*, (c) *c-Kit* mRNA expression in breast cancer and paired adjacent normal tissues form TCGA database. RFS of (d) *ATG2B*, (e) *BCL2L11* and (f) *c-Kit* gene expression and OS of (g) *ATG2B*, (h) *BCL2L11* and (i) *c-Kit* gene expression among breast cancer patients from Kaplan Meier Plotter.

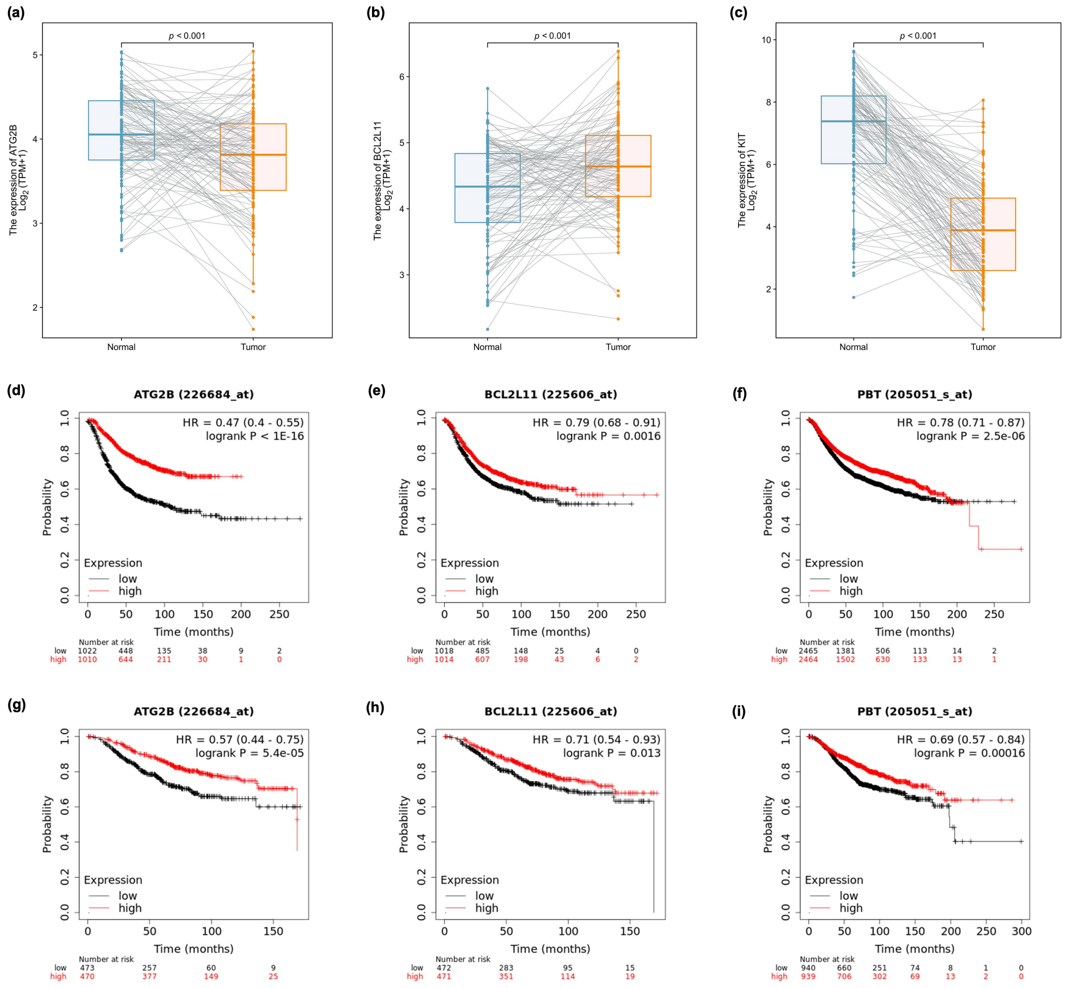

Abbreviations: TCGA, The Cancer Genome Atlas; RFS, recurrence free survival; OS, overall survival.
