# Supplementary material for: Additional prognostic value of polymorphisms within the 3′-untranslated region of programmed cell death pathway genes in early-stage breast cancer
Source: Front Immunol. 2024 Apr 16;15:1284579. doi: 10.3389/fimmu.2024.1284579 (PMC11058218; doi:10.3389/fimmu.2024.1284579)
Supplement: Supplementary file 2 [file Table_1.doc]

**Table S1 Basic information for candidate SNPs.**

| **Genes** | **dbSNP ID** | **Variants** | **Location ^a^** | **Binding MiRNAs** | **Energy change**  **(Kcal/mol)** | **MAF**  **in cases** | **Genotyping**  **rate** |
| --- | --- | --- | --- | --- | --- | --- | --- |
| CDKN1A | rs1059234 | C/T | Chr6:36653597 | miR-509-5p | -2 | 50.4% | 99.8% |
| E2F1 | rs3213180 | C/G | Chr20:32263624 | miR-1182 | -31.3 | 33.8% | 100% |
| BCL2L11 | rs6753785 | T/G | Chr2:111923630 | miR-556-5p | -6.7 | 41.4% | 100% |
| CDKN2A | rs3088440 | A/G | Chr9:21968159 | miR-663b | -2.3 | 12.1% | 99.9% |
| BMF | rs8116 | T/C | Chr15:40380207 | miR-582-5p | -13.8 | 45.2% | 99.9% |
| TP53INP1 | rs2459965 | A/G | Chr8:95940800 | miR-3065-5p | -12.5 | 11.7% | 100% |
|  |  |  |  | miR-545 | 2.4 |  |  |
| PDCD4 | rs1052550 | C/T | Chr10:112658027 | miR-3613-3p | -18.6 | 12.9% | 99.7% |
| PTEN | rs701848 | C/T | Chr10:89726745 | miR-1304 | 2.7 | 41.8% | 100% |
| NOTCH3 | rs12082 | C/T | Chr19:15270636 | miR-4268 | -2 | 11.0% | 100% |
| TP63 | rs35592567 | C/G | Chr3:189614636 | miR-140-5P | -22.4 | 17.4% | 100% |
| c-Kit | rs2213181 | C/T | Chr4:55604872 | miR-3157 | 24.3 | 8.2% | 100% |
| SIRT1 | rs4746720 | C/T | Chr10:69676830 | miR-561 | -13.7 | 42.8% | 99.8% |
| LATS2 | rs9552315 | T/C | Chr13:21547984 | miR-4267 | -19.9 | 48.3% | 100% |
| CDK6 | rs2285332 | C/G | Chr7:92241633 | miR-412 | 22.1 | 30.0% | 97.7% |
| PDK1 | rs1530865 | G/C | Chr2:173461090 | miR-3916 | 10.8 | 21.5% | 99.9% |
| EGFR | rs884225 | T/C | Chr7:55274084 | miR-486-3p | -12 | 47.5% | 99.9% |
|  |  |  |  | miR-3180-3p | 2.2 |  |  |
|  |  |  |  | miR-3196 | 2.2 |  |  |
|  |  |  |  | miR-3180 | 2.2 |  |  |
| BRIC5 | rs2239680 | T/C | Chr17:76219783 | miR-335 | -19.3 | 23.6% | 100% |
|  | rs1042542 | C/T | Chr17:76221428 | miR-4325 | -17.4 | 36.0% | 99.9% |
|  | rs4789560 | C/T | Chr17:76221462 | miR-542-3p | -3.9 | 40.6% | 99.9% |
| MDM4 | rs4252745 | C/G | Chr1:204519187 | miR-494 | -12.2 | 26.7% | 100% |
| ATG2B | rs7150025 | T/G | Chr14:96748288 | miR-940 | 19.6 | 23.8% | 99.8% |
|  | rs4900321 | A/T | Chr14:96749381 | miR-1256 | 2.1 | 17.8% | 100% |
| UVRAG | rs73500020 | G/T | Chr11:75854159 | miR-345 | -26.6 | 15.8% | 99.7% |
|  |  |  |  | miR-338-5p | 19.6 |  |  |
|  |  |  |  | miR-340 | 2.2 |  |  |
| ATG16L1 | rs6861 | C/T | Chr2:234204113 | miR-3622a-3p | -28.5 | 52.2% | 99.8% |
|  |  |  |  | miR-3622b-3p | -28.4 |  |  |
| ULK2 | rs205108 | C/A | Chr17:19678308 | miR-548a | -11.5 | 38.5% | 99.9% |
|  | rs205107 | G/A | Chr17:19678475 | miR-369-3p | 14.3 | 24.4% | 99.9% |
| HDAC5 | rs375171 | G/A | Chr17:42154470 | miR-620 | -23.7 | 18.5% | 99.9% |
|  |  |  |  | miR-1270 | -27.7 |  |  |
|  |  |  |  | miR-4308 | -3.8 |  |  |
|  |  |  |  | miR-4254 | -17.6 |  |  |
|  |  |  |  | miR-4292 | 2.1 |  |  |
|  | rs72822657 | A/G | Chr17:42155408 | miR-939 | 7 | 21.4% | 99.8% |
|  |  |  |  | miR-1225-3p | 34.5 |  |  |
|  |  |  |  | miR-4323 | 21.2 |  |  |

Abbreviations: MAF, minor allele frequency.

^a^ Genome Reference Consortium Human, build 37 (http://genome.ucsc.edu/cgi-bin/hgGateway).

Coding annotations for these SNPs were retrieved from the 1000 genomes (http://browser.1000genomes.org/index.html) and dbSNP (http://www.ncbi.nlm.nih.gov/SNP/).
