# Supplementary material for: Additional prognostic value of polymorphisms within the 3′-untranslated region of programmed cell death pathway genes in early-stage breast cancer
Source: Front Immunol. 2024 Apr 16;15:1284579. doi: 10.3389/fimmu.2024.1284579 (PMC11058218; doi:10.3389/fimmu.2024.1284579)
Supplement: Supplementary file 3 [file Table_2.doc]

**Table S2** Stratified analysis for associations between SNPs and EBC survival.

| **SNPs** | **Variables** | **iDFS** | | **DDFS** | | **BCSS** | | **OS** | |
| --- | --- | --- | --- | --- | --- | --- | --- | --- | --- |
|  |  | **Adjusted HR^a^** | ***P* value^b^** | **Adjusted HR^a^** | ***P* value^b^** | **Adjusted HR^a^** | ***P* value^b^** | **Adjusted HR^a^** | ***P* value^b^** |
| rs4789560 | Age at diagnosis |  |  |  |  |  |  |  |  |
|  | ≤ 35 | 1.065 (0.656-1.728) | 0.256 | 1.464 (0.863-2.484) | 0.029 | 1.722 (0.909-3.262) | 0.018 | 1.871 (0.988-3.544) | 0.008 |
|  | > 35 | 0.783 (0.632-0.971) |  | 0.771 (0.618-0.964) |  | 0.752 (0.588-0.961) |  | 0.743 (0.584-0.946) |  |
| rs2285332 | Grade |  |  |  |  |  |  |  |  |
|  | I + II | 1.084 (0.749-1.568) | 0.034 | 0.995 (0.671-1.474) | 0.024 | 0.758 (0.467-1.232) | 0.007 | 0.820 (0.517-1.302) | 0.013 |
|  | III | 2.364 (1.275-4.383) |  | 2.383 (1.249-4.548) |  | 2.477 (1.215-5.050) |  | 2.391 (1.174-4.869) |  |
| rs205107 | Grade |  |  |  |  |  |  |  |  |
|  | I+II | 0.815 (0.475-1.397) | 0.034 | 0.595 (0.316-1.123) | 0.027 | 0.868 (0.472-1.596) | 0.030 | 0.846 (0.461-1.555) | 0.035 |
|  | III | 2.258 (1.043-4.889) |  | 1.844 (0.848-4.011) |  | 2.621 (1.186-5.790) |  | 2.479 (1.126-5.459) |  |
| rs4900321 | HER2 |  |  |  |  |  |  |  |  |
|  | Negative | 1.146 (0.902-1.457) | 0.040 | 1.204 (0.939-1.545) | 0.016 | 0.984 (0.739-1.309) | 0.009 | 1.034 (0.782-1.366) | 0.012 |
|  | Positive | 1.747 (1.266-2.413) |  | 2.013 (1.442-2.809) |  | 1.827 (1.269-2.630) |  | 1.860 (1.296-2.669) |  |
| rs7150025 | HER2 |  |  |  |  |  |  |  |  |
|  | Negative | 0.878 (0.694-1.112) | 0.005 | 0.836 (0.652-1.072) | 0.022 | 0.935 (0.709-1.232) | 0.011 | 0.907 (0.691-1.191) | 0.017 |
|  | Positive | 0.472 (0.330-0.676) |  | 0.496 (0.341-0.721) |  | 0.493 (0.327-0.744) |  | 0.500 (0.333-0.751) |  |

Abbreviations: ^a^ Adjusted for age at diagnosis, tumor size, lymph node involvement, grade, hormone receptor, HER2 status, exception for stratification factor.

^b^ Heterogeneity test for differences between groups.
