# Supplementary material for: Additional prognostic value of polymorphisms within the 3′-untranslated region of programmed cell death pathway genes in early-stage breast cancer
Source: Front Immunol. 2024 Apr 16;15:1284579. doi: 10.3389/fimmu.2024.1284579 (PMC11058218; doi:10.3389/fimmu.2024.1284579)
Supplement: Supplementary file 5 [file Table_4.docx]

**Table S3** Function prediction for the rs4900321, rs7150025, rs6753785 and rs2213181.

| SNP | Gene | RegDB^1^ | Haploreg v4.2^2^ | | | | | |
| --- | --- | --- | --- | --- | --- | --- | --- | --- |
|  |  |  | Promoter  histone marks | Enhancer  histone marks | DNAse | Motifs  changed | Selected eQTL  hits | dbSNP  func annot |
| rs4900321 | ATG2B | 1f | LIV | ESDR, IPSC | -- | Sox | 26 hits | 3'-UTR |
| rs7150025 | ATG2B | 1f | LIV | 5 tissues | BRN | -- | 7 hits | 3'-UTR |
| rs6753785 | BCL2L11 | 1f | -- | SKIN, MUS | -- | GR | 1 hit | 3'-UTR |
| rs2213181 | KIT | 7 | -- | SKIN | -- | -- | -- | 3'-UTR |

Abbreviations: dbSNP func annot, dbSNP function annotation.

^1^ RegulomeDB: http://regulomedb.org/

^2^ Haploreg: https://pubs.broadinstitute.org/mammals/haploreg/haploreg.php
